# Pulmonary Complications Secondary to Immune Checkpoint Inhibitors

**DOI:** 10.1155/2020/4928648

**Published:** 2020-04-23

**Authors:** Hasan Ahmad Hasan Albitar, Narjust Duma, Konstantinos Leventakos, Alice Gallo De Moraes

**Affiliations:** ^1^Department of Internal Medicine, Mayo Clinic, Rochester, Minnesota, USA; ^2^Division of Medical Oncology, Mayo Clinic, Rochester, Minnesota, USA; ^3^Division of Pulmonary and Critical Care, Mayo Clinic, Rochester, Minnesota, USA

## Abstract

**Background:**

Immune checkpoint inhibitors (ICI) have changed the landscape in the treatment of a number of cancers. Immune-related adverse events (irAEs) have emerged as a serious clinical problem with the use of ICI.

**Methods:**

All oncology patients diagnosed with pulmonary complications secondary to ICI at Mayo Clinic Rochester from January 1, 2012 to December 31, 2018 were reviewed. Demographics, comorbidities, smoking, and oncologic history were analyzed.

**Results:**

A total of 10 patients developed pulmonary complications secondary to ICI. Seven patients were men (70%), and the median age at diagnosis was 61.5 (IQR 55.8-69.3) years. All patients had stage IV disease. Melanoma was the most common malignancy. Seven (70%) patients had a positive smoking history, and 6 (60%) were obese (BMI > 30). Most cases were grade 2 pneumonitis (70%). One patient with grade 4 pneumonitis required endotracheal intubation and a prolonged course of systemic corticosteroids (>30 days). Eight (80%) patients received prior radiation therapy. The median time from initiation of ICI to pneumonitis diagnosis was 3.5 months.

**Conclusion:**

Melanoma was the most common malignancy, the majority of patients had grade 2 pneumonitis and required treatment with steroids, and all patients affected by ICI-related pneumonitis had stage IV malignancy. Potential risk factors included smoking history, prior radiotherapy, obesity, and advance stage at the time of ICI initiation. Extrapulmonary irAEs are common in patients with pneumonitis.

## 1. Introduction

Programmed death 1 (PD-1) and its ligands (PD-L1 and PD-L2), in addition to cytotoxic T-lymphocyte-associated protein 4 (CTLA-4), are negative regulators of T-cell activation that play an integral role in immune homeostasis [1, 2]. The development of pharmaceutical anti-PD-1 and PD-L1 antibodies and monoclonal antibodies targeting CTLA-4 has changed the landscape in the treatment of a number of cancers and improved survival from months to complete remission in some cases [3]. However, with the development of these novel agents came a new group of distinctive immune adverse reactions, thought to be related to cytokine release, that range from transient and benign to severe and fatal [4, 5]. They are referred to as immune-related adverse events (irAEs). Evidence shows that immune checkpoint inhibitor (ICI) use is associated with increased risk of all-grade pneumonitis compared with other conventional chemotherapeutic agents [6]. Pulmonary irAEs are of special interest because they can lead to intensive care unit (ICU) admission, endotracheal intubation, and in severe cases, death.

**Figure 1:**
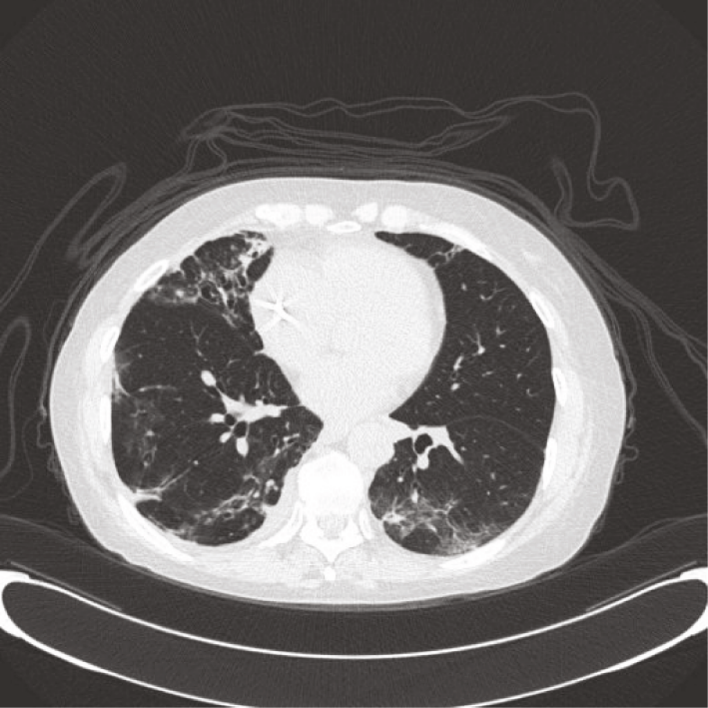
Chest computed tomography example of a case with immune-checkpoint inhibitor induced pneumonitis showing patchy bilateral areas of consolidation and ground-glass attenuation that appeared following initiation of ICI.

Commonly encountered computed tomography findings include bilateral consolidative changes and ground-glass opacities (Figure 1), predominantly in peripheral distribution but also with interlobular septal thickening in basilar distribution [7]. However, imaging findings are nonspecific and distinguishing ICI-pneumonitis from radiation-induced pneumonitis and pulmonary infections can be challenging. The cessation of ICI therapy alone is sufficient in mild pneumonitis cases and corticosteroids are typically used for treatment of more severe, symptomatic cases [8, 9]. Most irAEs respond to corticosteroids and resolve within 3 months [10].

Our objective in the present study is to present our center's clinical experience with ICI-induced pneumonitis, to report the baseline patient characteristics in 10 patients with ICI-induced pneumonitis and to compare the rate of these complications with the data published in previous reports.

## 2. Materials and Methods

### 2.1. Patients

Study inclusion criteria specified patient age greater than 18 years; histologically confirmed diagnosis of solid malignancy for which treatment with an ICI is approved by the US Food and Drug Administration; more than 3 months follow-up at Mayo Clinic in Rochester, Minnesota; and receipt of at least 1 dose of ICIs. Patients with hematologic malignancy, those without research consent, and patients with no close follow-up at Mayo Clinic in Rochester were all excluded.

### 2.2. Data Collection

Using the electronic medical record system, we identified patients with ICI-induced pneumonitis at Mayo Clinic's Rochester campus from January 1, 2012 to December 31, 2018. This study was approved by the Mayo Clinic's Institutional Review Board. Cases were reviewed by at least 1 radiologist and 1 pulmonologist and were classified and graded according to the National Cancer Institute Common Terminology Criteria for Adverse Events version 4.0 (Table 1) [11, 12].

## 3. Results

### 3.1. Baseline Patient Characteristics

Ten patients with median age of 61.5 (IQR 55.8-69.3) were identified. Baseline characteristics are summarized in Table 2.

### 3.2. Primary Malignancy of Patients with ICI-Induced Pneumonitis

Melanoma was the most common malignancy (*n* = 5, 50%) followed by small cell lung cancer (SCLC) (*n* = 1, 10%), spindle cell carcinoma (*n* = 1, 10%), neuroendocrine tumor of the epiglottis (*n* = 1, 10%), lung adenocarcinoma (*n* = 1, 10%), and Merkel cell carcinoma (*n* = 1, 10%). All patients with pneumonitis had stage IV cancer at the time immunotherapy was introduced. The most common sites of metastasis were liver and bones, seen in 4 patients (40%) and 6 patients (60%), respectively.

### 3.3. ICI Use and Pneumonitis Grade

Immune checkpoint inhibitors at the time of pneumonitis were pembrolizumab (*n* = 5, 45.5%), nivolumab (*n* = 3, 27.3%), ipilimumab (*n* = 2, 18.2%), and atezolizumab (*n* = 1, 9%), and 1 patient was on dual therapy with ipilimumab and nivolumab. Two patients (20%) had grade 1 pneumonitis; 7 (70%) had grade 2; and 1 (10%) had grade 4 pneumonitis.

### 3.4. Other irAEs Encountered with ICI-Induced Pneumonitis

In addition to pneumonitis, 6 patients (60%) had other irAEs. The most commonly encountered nonpulmonary irAEs were autoimmune hepatitis and colitis. Data about other irAEs are summarized in Table 3.

### 3.5. Outcomes after ICI Treatment

Five patients (50%) had a response after initiation of ICI therapy, 3 (30%) had disease progression, and 2 (20%) had mixed response. The median (IQR) time from initiation of ICI treatment to the pneumonitis diagnosis was 3.5 (range; 1.5-28) months. BAL was performed for 4 patients (40%) and showed inflammatory macrophage-predominant alveolitis in 3 (75%) patients. Microbiologic studies on BAL specimens for viruses, bacteria, fungi, and parasites were negative among all patients who underwent BAL.

Eight patients (80%) required systemic corticosteroids for the treatment of pulmonary and/or extrapulmonary irAEs. ICI treatment was discontinued for 8 patients (80%) and was resumed and continued for 5 (50%) after the pneumonitis cleared. One patient with grade 1 pneumonitis was continued on ICI without interruption or steroids, and 1 with grade 2 pneumonitis was treated with drug holding only. All other patients were treated with systemic corticosteroids with prednisone being the main corticosteroid used. The duration of the treatment with corticosteroids ranged from 30 to 360 days. The patient with grade 4 pneumonitis required endotracheal intubation and a prolonged course of corticosteroids (>30 days). None of the patients received any immunosuppressive treatment other than the corticosteroids.

**Figure 2:**
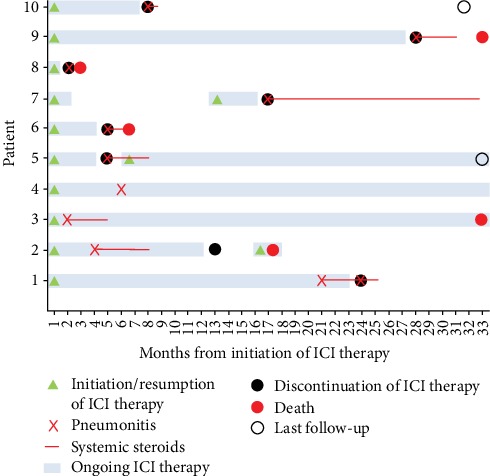
Timeline of ICI therapy, pneumonitis, steroid therapy, and follow-up.

During follow-up, 5 patients (50%) died. Early mortality rate within 1 month of pneumonitis was 10%. Among the patients who died, 1 had grade 4, and 4 had grade 2 pneumonitis. Among the 5 patients still alive at study completion, 3 patients had grade 2 and 2 patients had grade 1 pneumonitis. Cause of death was directly related to pneumonitis in 1 patient only. Details are summarized in Figure 2.

## 4. Discussion

The reported overall incidence of all-grade pneumonitis has ranged from 1.3% to 11% [13–16]. According to Nishino et al. [16], patients with pneumonitis most commonly present with cough (60%), dyspnea (55%), and less frequently with fever. The diagnosis of pneumonitis may represent a challenge to physicians, especially because of the similarity in presentation between ICI-induced pneumonitis, radiation pneumonitis, pneumonia, and malignant lung infiltration [17].

In this study, we presented our center's experience with ICI-induced pneumonitis and found that all patients had stage IV malignancy, melanoma was the most common malignancy, the majority of cases were grade 2 pneumonitis and that most were treated with steroids. We also found an early mortality rate of 10% which was comparable to that observed by Delaunay et al. [18] (9.4%). However, both studies showed a lower mortality rate than previous studies that reported a mortality rate of 30% to 36% [19–21]. Our study's lower mortality rate could be related to increased physician awareness and early detection and treatment of pneumonitis compared to the earlier studies and could be confounded by the low number of patients.

We also report a median time between initiation of ICI therapy and development of pneumonitis of 3.5 months, which is comparable to prior studies. According to Brahmer et al. [14], the median time to onset of treatment-related pneumonitis was 15.1 weeks. According to Borghaei et al. [22], the median time to onset of pulmonary events was 31.1 weeks. Fujimoto et al. [19] observed a median time of 1.3 months between the start of systemic anticancer treatment and the onset of pneumonitis. Similarly, Delaunay et al. [18] showed that most of their study's pneumonitis cases occurred during the first months of treatment, with a median time to onset of 2.3 months. Naidoo et al. [23] reported that the median time to onset of pneumonitis was 2.8 months.

An interesting finding in our study was that a substantial percentage of study patients with pneumonitis (60%) were obese; however, causality cannot be established using the current study design and the small number of patients. Investigators have previously shown that adipose tissue produces and releases various proinflammatory factors, including cytokines such as tumor necrosis factor-*α* and interleukin-6 (IL-6) [24]. Evidence also reports a positive correlation between serum IL-6 levels and body fat mass [25]. Moreover, tumor necrosis factor-*α* is overexpressed in the adipose tissue of obese humans [26, 27]. These cytokines trigger the production of acute-phase reactants, such as C-reactive protein, plasminogen activator inhibitor-1, and serum amyloid-A [28]. Therefore, obesity has been considered a proinflammatory state because of the increased level of inflammatory cytokines [29].

The present study has limitations. First, we recognize that the study has a small patient cohort. However, the small number of patients identified in this study is related to the fact that most patients receive their treatment at facilities elsewhere and visit Mayo Clinic for a second opinion or for restaging scans every 3 months only. This schedule limits the quality of data and decreases the possibility of recording the rate of pulmonary complications accurately because of no access to the outside records. Second, the retrospective design limits the study. However, our objective was to report our center's experience with ICI-induced pneumonitis and to report the characteristics of patients who developed pneumonitis, for which a retrospective design is adequate. Third, the diagnosis of pneumonitis was determined on the basis of expert opinion after the review of imaging findings. We acknowledge that attributing causality to ICI use is always a challenge because of the lack of universal diagnostic criteria and gold standard diagnostic testing. Yet, we performed a battery of tests—including but not limited to bronchoscopy with BAL—to rule out other causes such as infections and tumor infiltration of the lungs. BAL testing was performed for 40% of our patients. This percentage is in contrast to prior studies, which did not include BAL testing in the evaluation of suspected pneumonitis cases [20, 29]. Moreover, the clinical, laboratory, radiographic, and pathologic characteristics of the study patients were reviewed extensively by experts in the field. Lastly, although we report on multiple findings in patients with pneumonitis in this study including the high rate of obesity, causality cannot be established given the current study design.

## 5. Conclusions

Melanoma was the most common malignancy associated with pneumonitis, the majority of patients had grade 2 pneumonitis and required treatment with steroids, and all patients affected by ICI-related pneumonitis had stage IV malignancy. Potential risk factors included smoking history, prior radiotherapy, obesity, and advance stage at the time of ICI initiation. Extrapulmonary irAEs are common in patients with pneumonitis.

## Figures and Tables

**Table 1:** Grades of CTCAE version 4.0.

CTCAE grade	Clinical presentation
1	Asymptomatic pneumonitis; clinical or diagnostic observations only
2	Symptomatic pneumonitis; medical intervention indicated; limited instrumental ADL
3	Severe symptoms secondary to pneumonitis; limited self-care ADL; oxygen treatment indicated
4	Life-threatening respiratory compromise; urgent intervention indicated (e.g., tracheotomy and intubation)
5	Death secondary to pneumonitis

Abbreviations: ADL: activities of daily living; CTCAE: Common Terminology of Criteria for Adverse Events. Modified from National Cancer Institute ^12^.

**Table 2:** Baseline characteristics of the 10 patients with pneumonitis secondary to ICI use.

Baseline characteristics	Patients, no. (%)
Age (y)	
≥65	4 (40)
<65	6 (60)
Male sex	7 (70)
Body mass index >30	6 (60)
Positive smoking history (current and prior use)	7 (70)
Cancer stage IV at time of pulmonary complications	10 (100)
Site of metastasis	
Liver	4 (40)
Bone	6 (60)
Charlson comorbidity index	
0-8	4 (40)
9-17	6 (60)
Previous radiation history	8 (80)
Chemotherapy before initiation of ICI use	
Yes	7 (70)
No	3 (30)
Medical history of COPD or emphysema	
Yes	2 (20)
No	8 (80)
Use of corticosteroid inhaler at time of pneumonitis	
Yes	1 (0)
No	9 (90)

Abbreviations: COPD: chronic obstructive pulmonary disease; ICI: immune checkpoint inhibitor.

**Table 3:** Extrapulmonary irAEs^∗^.

Other irAEs	Patients, no. (%)	Grade	Timing in relation to pneumonitis
Encountered	6 (60)		
Not encountered	4 (40)		
Type			
Hepatitis	3 (33.3)	1, 2, 1	Concurrent in all 3 cases
Colitis	2 (22.2)	2, 2	Concurrent in both cases
Sjögren syndrome	1 (11.1)	-	Prior to
Neutropenia	1 (11.1)	-	Prior to
Pericarditis and pericardial effusion	1 (11.1)	2	Concurrent
Conjunctivitis	1 (11.1)	1	Concurrent

Abbreviation: irAE: immune-related adverse event. ^∗^Some patients had more than one extrapulmonary irAE.

## Data Availability

The data used to support the findings of this study are available from the corresponding author upon request.

## Abbreviations

BAL: Bronchoalveolar lavage
COPD: Chronic obstructive pulmonary disease
ICI: Immune checkpoint inhibitor
ICU: Intensive care unit
IL-6: Interleukin-6
IQR: Interquartile range
irAE: Immune-related adverse event
SCLC: Small cell lung cancer
PD-1: Programmed death 1.
